# Oral Transmission of L-type Bovine Spongiform Encephalopathy in Primate Model

**DOI:** 10.3201/eid1801.111092

**Published:** 2012-01

**Authors:** Nadine Mestre-Francés, Simon Nicot, Sylvie Rouland, Anne-Gaëlle Biacabe, Isabelle Quadrio, Armand Perret-Liaudet, Thierry Baron, Jean-Michel Verdier

**Affiliations:** Institut National de la Santé et de la Recherche Médicale (INSERM) U710, Montpellier, France (N. Mestre-Francés, S. Rouland, J.-M. Verdier);; Université Montpellier 2, Montpellier (N. Mestre-Francés, S. Rouland, J.-M. Verdier);; École Pratique des Hautes Etudes, Paris, France (N. Mestre-Francés, S. Rouland, J.-M. Verdier);; Agence Nationale de Sécurité Sanitaire, Lyon, France (S. Nicot, A.-G. Biacabe, T. Baron);; Hopitaux Civils de Lyon, Lyon, France (I. Quadrio, A. Perret-Liaudet);; Université Lyon 1, Lyon (I. Quadrio, A. Perret-Liaudet);; INSERM U1028, Lyon (I. Quadrio, A. Perret-Liaudet);; Centre National de la Recherche Scientifique, Lyon (I. Quadrio, A. Perret-Liaudet)

**Keywords:** prion, prions and related diseases, bovine spongiform encephalopathy, BSE, L-type BSE, mouse lemur, primate, non-human primate model, oral transmission, cattle

## Abstract

We report transmission of atypical L-type bovine spongiform encephalopathy to mouse lemurs after oral or intracerebral inoculation with infected bovine brain tissue. After neurologic symptoms appeared, transmissibility of the disease by both inoculation routes was confirmed by detection of disease-associated prion protein in samples of brain tissue.

Transmissible spongiform encephalopathies, also known as prion diseases, are fatal neurodegenerative disorders that affect humans and animals. An atypical form of bovine spongiform encephalopathy (BSE) was recently identified in cattle in Europe (*1**,**2*), North America (*3*), and Japan (*4*). This atypical BSE was designated L-type BSE (L-BSE) because Western blot analysis showed that the disease-associated protease-resistant prion protein (PrP^res^) was of lower apparent molecular mass than in the agent of classical BSE, which is involved in the major foodborne epizooty in cattle and in variant Creutzfeldt-Jakob disease in humans (*5*).

Evidence from experimental studies in primate models (*6**,**7*) and transgenic mice expressing human prion protein (PrP) (*8**,**9*) suggests that the rare and putatively sporadic form of L-BSE (*10*) presents a higher risk than classical BSE for transmission to humans. However, a major unresolved issue is whether L-BSE can be transmitted by the oral route. To address this issue, we inoculated gray mouse lemurs (*Microcebus murinus*), a nonhuman primate model, by the oral and intracerebral (IC) routes with the agent of L-BSE.

## The Study

A total of 12 mouse lemurs of both sexes (Center for Breeding and Experimental Conditioning of Animal Models, University Montpellier 2, Montpellier, France) were maintained in animal Biosafety Level 3 facilities, according to requirements of the French ethics committee (authorization CE-LR-0810). Young and adult lemurs were fed (8 animals) or IC inoculated (4 animals) with 5 or 50 mg of L-BSE–infected brain tissue (10% homogenate in 5% glucose) (Table). The isolate for the L-BSE agent (02–2528) was derived from cattle in France (*11*). When progression of prion disease was evident, the lemurs were euthanized and their brains were isolated. Brains were processed for Western blot analysis with SHa31 monoclonal antibody against PrP for PrP^res^ detection, as described in mice (*11*); for histologic examination by using hematoxylin and eosin staining; and for disease-associated prion protein (PrPd) immunochemical detection by using the paraffin-embedded tissue blot method or immunohistochemical analysis with monoclonal antibody 3F4 against PrP.

**Table:** Experimental transmission of cattle-derived L-BSE agent to 12 mouse lemurs, by 2 routes of inoculation*

Inoculation route	L-BSE dose, mg	Inoculated animals			
		No. inoculated (no. alive)	Age at inoculation	Survival after inoculation, mo	Positive for PrPd †
Intracerebral	5	4	1 y	19; 19.5; 22; 22	4/4
Oral	50	3 (1‡)	2 mo or 2 y	18‡; 32	2/2
Oral	5	5 (2‡)	2 mo or 2 y	27; 33; 34	2/3

*L-BSE, L-type bovine spongiform encephalopathy source; PrPd, disease-associated prion protein. Source of L-BSE, 02–2528. †Results obtained by Western blot analysis and/or paraffin-embedded tissue-blot analysis and/or immunohistochemical analysis. ‡Animals were inoculated at 2 y of age.

Beginning ≈3 months before the terminal stage of the disease (19–22 months after inoculation), neurologic symptoms developed in the 4 mouse lemurs that received IC inoculations (Table). In all 4 animals, initial clinical signs and symptoms were blindness, thigmotaxic behavior, and poor appearance of the fur. Appetite and general fitness were maintained; anxiety and aggressiveness were not observed. Next, locomotion became slower, followed by incoordination and loss of balance in the last month of life. Ipsilateral circling behavior was reported, indicating unilateral degeneration of the striatum. This behavior stopped 15 days after onset, suggesting damage to the contralateral striatum. Disequilibrium, with frequent falls, became more noticeable. At the terminal stage of the disease, the animals were prostrate.

One orally inoculated lemur, which was fed 5 mg of infected brain and euthanized 27 months later, had signs and symptoms of disease similar to those in IC-inoculated animals, except for the ipsilateral circling behavior. In 2 lemurs fed 50 mg and 2 others fed 5 mg of L-BSE–infected brain, clinical signs and symptoms of prion disease developed just a few weeks before the animals were euthanized (18 and 32 months and 33 and 34 months after inoculation, respectively). Disease was characterized by progressive prostration, loss of appetite, and poor appearance of the fur, without incoordination or disequilibrium. The 3 remaining lemurs were orally inoculated at 2 years of age and were still alive and healthy 28 months after inoculation (Table).

**Figure 1:**
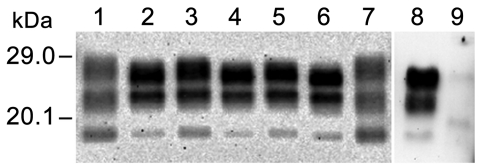
Western blot analysis of protease-resistant prion protein in the brain (thalamus/hypothalamus) and spleen of mouse lemurs inoculated with a cattle-derived L-type bovine spongiform encephalopathy (BSE) isolate by oral and intracerebral routes by using SHa31 monoclonal antibody against prion protein. Lanes 1, 7: cattle L-type BSE isolate (02-2528); lanes 2, 3: brain sample from intracerebral inoculation at 5 mg; lane 4: brain sample from oral inoculation at 50 mg; lanes 5, 6: brain sample from oral inoculation at 5 mg; lanes 8, 9: spleen samples from oral inoculation at 5 mg, positive and negative, respectively.

PrP^res^ was readily detected by Western blot analysis in brain extracts (thalamus/hypothalamus region) from 8 of the 9 animals examined (Table), although at lower levels in the lemur that was euthanized earlier (i.e., 18 months after inoculation). Western blot analyses showed uniform PrP^res^ molecular profiles, irrespective of the route or dose of inoculation, with a low apparent molecular mass (≈19 kDa, similar to the PrP^res^ in the original cattle brain) (Figure 1). However, the PrP^res^ profile in mouse lemurs was characterized by a higher proportion of di- and monoglycosylated species (>95% of the total signal) than in the inoculum of the agent of bovine L-BSE (≈80%). In addition, PrP^res^ was detected by Western blot in the spleens of 3 (1 IC inoculated and 2 fed with 5 mg of cattle brain) of the 9 animals examined (Figure 1).

**Figure 2:**
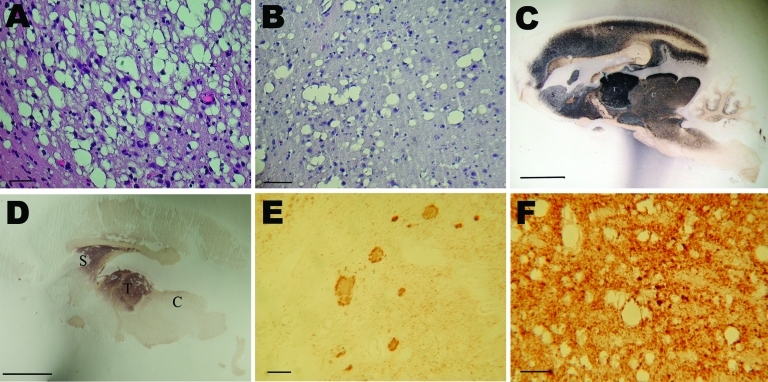
Histopathologic and disease-associated prion protein (PrPd) immunodetection in the brain of 2 mouse lemurs after intracerebral (5 mg) or oral (50 mg) inoculation with a cattle-derived L-type bovine spongiform encephalopathy isolate. A, B) Spongiosis in the striatum; scale bars = 30 μm. C, D) Paraffin-embedded tissue blot analysis of sagittal brain section; scale bars = 500 μm. E, F) PrPd immunodetection; scale bars = 30 μm. Analyses in C–F were performed by using the 3F4 monoclonal antibody against PrP. C, colliculus; S, striatum; T, thalamus.

Histopathologic analysis showed severe spongiform changes in the brains of the 4 IC-inoculated mouse lemurs (Figure 2, panel A). The brains displayed a pattern of vacuolation characterized by intense spongiosis with many confluent vacuoles in the basal telencephalon (septum, striatum, caudate putamen nuclei), midbrain (thalamus, hypothalamus), mesencephalon (colliculi), and in some parts of the brainstem (tegmental ventral area, raphe nuclei). Lesions in the cortex and hippocampus were less severe than in the subcortical areas. Cerebellum showed occasional small-size vacuoles. Among the 5 orally inoculated animals, 2 (1 fed 5 mg, the other fed 50 mg) showed histopathologic features similar to those observed in IC-inoculated animals. In the other 3 orally inoculated animals, spongiosis was characterized by fewer vacuoles and was restricted to the striatum (Figure 2, panel B), thalamus, colliculi, and brainstem.

Distribution of PrPd in the brain was assessed by paraffin-embedded tissue blot (Figure 2, panels C and D) or immunohistochemical analysis with 3F4 antibody (Figure 2, panels E and F). Results for IC-inoculated animals showed that PrPd strongly accumulated in a dense synaptic pattern associated with nonamyloid plaques in the striatum, several thalamic nuclei (Figure 2, panel E), the external cortex of the colliculi, and the tegmental area. Other areas that were slightly less affected (e.g., neocortex and hippocampus) showed few coarse granules and synaptic deposits. The cortical molecular layer and the corpus callosum were devoid of PrPd (Figure 2, panel C). In orally inoculated animals, PrPd was strongly accumulated in the striatum and thalamus (Figure 2, panel D) but weakly accumulated in the cortex. Immunohistochemical analysis showed synaptic deposits (Figure 2, panel F), and some focal deposits were evident in animals that survived longer. No plaques were detected in orally inoculated animals.

## Conclusions

We demonstrated that the agent of L-BSE can be transmitted by the oral route from cattle to mouse lemurs. As expected, orally inoculated animals survived longer than IC-inoculated animals. Orally inoculated lemurs had less severe clinical signs and symptoms, with no evidence of motor dysfunction. It was previously suggested that the agent of L-BSE might be involved in the foodborne transmission of a prion disease in mink (*11**,**12*), a species in which several outbreaks of transmissible mink encephalopathy had been identified, notably in the United States (*13*).

Our study clearly confirms, experimentally, the potential risk for interspecies oral transmission of the agent of L-BSE. In our model, this risk appears higher than that for the agent of classical BSE, which could only be transmitted to mouse lemurs after a first passage in macaques (*14*). We report oral transmission of the L-BSE agent in young and adult primates. Transmission by the IC route has also been reported in young macaques (*6**,**7*). A previous study of L-BSE in transgenic mice expressing human PrP suggested an absence of any transmission barrier between cattle and humans for this particular strain of the agent of BSE, in contrast to findings for the agent of classical BSE (*9*). Thus, it is imperative to maintain measures that prevent the entry of tissues from cattle possibly infected with the agent of L-BSE into the food chain.
